# Discovery of PAK2 as a Key Regulator of Cancer Stem Cell in Head and Neck Squamous Cell Carcinoma Using Multi-Omic Techniques

**DOI:** 10.1155/sci/1325262

**Published:** 2025-11-19

**Authors:** Puyu Wang, Shengshan Xu, Qian Guo, Yulin Zhao

**Affiliations:** ^1^Department of Otolaryngology Head and Neck Surgery, The First Affiliated Hospital of Zhengzhou University, Zhengzhou, Henan, China; ^2^Department of Thoracic Surgery, Jiangmen Central Hospital, Jiangmen, Guangdong, China

**Keywords:** cancer stem cells, PAK2, programed cell death, single-cell sequencing, spatial transcriptomics, tumor immune microenvironment

## Abstract

Head and neck squamous cell carcinoma (HNSC) is an aggressive malignancy whose progression is closely associated with dysregulation of programed cell death (PCD) pathways and cancer stem cell (CSC) characteristics. To systematically screen for key pathogenic genes, this study performed single-cell analysis on the GSE150321 dataset. The identified cell-specific genes were intersected with PCD- and CSC-related genes, yielding 24 candidate genes for preliminary screening. Further refinement using multiple machine learning (ML) algorithms identified PAK2 as the most central gene among these candidates. Analysis of TCGA and external datasets confirmed that PAK2 is significantly overexpressed in HNSC tissues, demonstrating good diagnostic value and strong association with poor patient prognosis. Functional studies revealed that PAK2 overexpression positively correlates with malignant phenotypes such as metabolic reprograming and tumor metastasis. Notably, PAK2 expression showed a significant negative correlation with antitumor immune status and negatively regulated the infiltration of multiple immune cell types. Spatial transcriptomics and single-cell sequencing analyses revealed PAK2's specific expression patterns within the tumor microenvironment, confirming its influence on the activity of immune-related molecules and immunomodulators. Finally, through Connectivity Map (cMAP) screening and molecular docking, we identified the small molecule compound butein as an effective agent capable of reversing PAK2-mediated procancer molecular features. Butein exhibits stable binding to the PAK2 protein, suggesting its potential as a targeted therapeutic agent. In summary, through multi-omics integration analysis, this study first reveals that PAK2 plays a central role in the pathogenesis of HNSC by regulating PCD, tumor stem cell properties, and the immune microenvironment, and provides a candidate drug for its targeted therapy.

## 1. Introduction

Head and neck squamous cell carcinoma (HNSC) continues to pose a significant threat as a deadly cancer type, exhibiting minimal progress in treatment options. This malignancy is notably marked by elevated rates of recurrence and a marked resistance to standard therapeutic approaches [1, 2]. Programed cell death (PCD), including apoptosis, necroptosis, and pyroptosis, serves as a critical safeguard against tumorigenesis by eliminating malignant cells and maintaining tissue homeostasis [3–5]. However, dysregulation of PCD pathways is a hallmark of HNSC progression, enabling cancer cells to evade immune surveillance and sustain proliferative signaling [6]. Despite the identification of key PCD-related genes (e.g., CASP3, MLKL), their clinical translation remains hindered by tumor heterogeneity and compensatory mechanisms [7, 8]. This underscores the urgent need to identify robust PCD regulators that orchestrate HNSC's lethal adaptability [9]. Furthermore, the tumor stem cell theory offers a novel perspective for explaining treatment resistance and recurrence in HNSC. Cancer stem cells (CSCs) constitute a subpopulation of cells possessing self-renewal and multipotent differentiation capabilities. Insensitive to conventional chemotherapy and radiotherapy, they are regarded as the “seeds” driving tumor initiation, progression, and metastasis.

Recent advances in single-cell and spatial transcriptomics have unveiled the intricate interplay between tumor microenvironment (TME) dynamics and PCD evasion [10, 11]. Notably, immune cells such as CD8+ T cells and tumor-associated macrophages modulate PCD susceptibility through cytokine secretion and checkpoint ligand expression [12]. However, systematic identification of PCD master regulators that integrate TME crosstalk remains elusive. Here, we employed a machine learning (ML) framework to dissect HNSC's PCD landscape, prioritizing PAK2—a serine/threonine kinase implicated in cytoskeletal remodeling—as a novel PCD hub [13]. PAK2's role in HNSC is paradoxical, its oncogenic hijacking in tumors remains unexplored.

To address these gaps, we integrated multi-omics datasets, including single-cell RNA sequencing (scRNA-seq), spatial transcriptomics, and clinical proteomics, with ML frameworks to systematically interrogate PAK2's role in HNSC pathogenesis. We hypothesized that PAK2 acts as a central hub linking PCD dysregulation, metabolic reprograming, and immune suppression in HNSC, and that its targeting could reverse oncogenic phenotypes [14]. This study aimed to: (1) identify PAK2 as a candidate biomarker through ML-driven analysis of scRNA-seq data; (2) validate its clinical relevance using multi-cohort transcriptomic and proteomic datasets; (3) decode its biological functions via pathway enrichment and metabolic gene set variation analysis (GSVA); (4) map its spatial distribution and immune correlates using spatial transcriptomics and immunogenomic profiling; and (5) nominate actionable inhibitors through drug repurposing and molecular docking.

Our approach leverages the complementary strengths of scRNA-seq to resolve cell type-specific PAK2 expression, spatial transcriptomics to localize its microenvironmental interactions, and ML to prioritize its role among PCD-related genes [15]. By integrating these modalities, we aimed to transcend the limitations of reductionist approaches and elucidate PAK2's multimodal contributions to HNSC pathogenesis [16, 17]. This study not only advances mechanistic understanding of PAK2 in HNSC but also establishes a paradigm for systems-level dissection of oncogenic drivers in the multi-omics era.

## 2. Materials and Methods

### 2.1. Single-Cell Transcriptomics

Tumor stem cell-related genes are sourced from the PathCards database (https://pathcards.genecards.org/) [18]. scRNA-seq data from patients diagnosed with HNSC, specifically sourced from the GEO database under the accession number GSE150321, were meticulously retrieved for analysis [19]. This dataset encompasses both the tumor core and the corresponding paired peripheral tissues, providing a comprehensive view of the cellular landscape. The data preprocessing and analytical procedures were performed using the Seurat package (version 4.0), a widely adopted tool for scRNA-seq analysis. Initially, the expression matrices underwent normalization through the NormalizeData function, ensuring that the data were appropriately scaled for subsequent analyses. Following normalization, dimensionality reduction was achieved using the Uniform Manifold Approximation and Projection (UMAP) method via the RunUMAP function, which facilitated the visualization of the high-dimensional data in a two-dimensional space. The results were then graphically represented using the DimPlot function, allowing for an intuitive interpretation of the cellular distributions. To further elucidate the biological differences between the tumor and adjacent tissues, differential gene expression analysis was performed using the FindMarkers function, which identified specific genes that exhibited significant expression variations, thereby shedding light on the molecular underpinnings of the tumor microenvironment.

### 2.2. Clinical and Diagnostic Evaluation

PAK2's diagnostic potential was thoroughly assessed using receiver operating characteristic (ROC) curves, a method that allows for the evaluation of the performance of a diagnostic test, facilitated by the pROC package, which is specifically designed for such analyses in R programing. The analysis was conducted with 95% confidence intervals to ensure the reliability and statistical significance of the results obtained, thereby reinforcing the credibility of the findings. Furthermore, model calibration and goodness-of-fit tests were meticulously performed to validate the accuracy of the predictions made by the model, ensuring that the model's predictions closely aligned with the actual observed outcomes. In addition, the differential expression of PAK2 between tumor and normal tissues was statistically evaluated across the extensive TCGA-HNSC cohort, which encompasses a wide range of NHSC samples, providing a comprehensive understanding of its role in cancer pathology and potentially illuminating its implications for targeted therapies and treatment strategies.

### 2.3. Functional Annotation

Samples were meticulously stratified into two distinct groups based on their PAK2 expression levels: the PAK2-high group, which comprised the top 30% of samples exhibiting elevated PAK2 expression, and the PAK2-low group, consisting of the bottom 30% of samples with significantly reduced PAK2 levels. To delve deeper into the biological implications of these differences, differential gene expression analysis was performed utilizing the limma package, a robust tool for identifying variations in gene expression profiles. Furthermore, KEGG pathway enrichment analysis was conducted using the clusterProfiler package, allowing for a comprehensive understanding of the metabolic pathways that may be influenced by PAK2 expression. The activity of various metabolic pathways was quantified through GSVA, providing a nuanced view of pathway dynamics. Additionally, functional state scores derived from 14 specific gene sets were computed through *z*-score normalization, enabling a standardized comparison of functional states across samples. These scores were then correlated with PAK2 expression levels using Pearson correlation, facilitating insights into the relationship between PAK2 expression and the functional states of the samples analyzed.

### 2.4. Immune Landscape Profiling

Immunogenicity scores, which include various parameters such as cytolytic activity and the presence of tertiary lymphoid structures, were meticulously calculated utilizing the user-friendly easier package, designed to streamline the analysis process. The groups categorized by high and low levels of PAK2 expression, determined through a median split, were then systematically compared to assess any significant differences in their immune scores. Furthermore, the correlation between tumor immune dysfunction (TIP) scores and PAK2 expression levels was evaluated using the Spearman correlation coefficient, providing insights into the relationship between these two critical factors in the context of tumor immunology.

### 2.5. Spatial Transcriptomics

Cellular deconvolution of the 10x Visium slides, which involved the integration of scRNA-seq reference data from the dataset GSE150321, was meticulously conducted to ensure accurate results. The quality control process was comprehensive, incorporating stringent thresholds for both gene and unique molecular identifier (UMI) counts, as well as assessments of mitochondrial RNA levels to filter out low-quality cells. To further enhance the analysis, cell type-specific signatures were identified and scored using Cottrazm, focusing on the top 25 marker genes that are characteristic of each cell type. The spatial enrichment of these signatures was then effectively visualized through the use of SpatialFeaturePlot in the Seurat package, allowing for a clear representation of the spatial distribution of different cell types across the tissue sections.

### 2.6. ML Workflow

Data were meticulously partitioned into training and test sets in a balanced ratio of 50:50 using the createDataPartition function from the caret package, ensuring that both subsets were representative of the overall dataset. Subsequently, various models, including least absolute shrinkage and selection operator (LASSO), support vector machine (SVM), and random forest (RF), were trained using the train function, allowing for the development of robust predictive algorithms. After training, these models were rigorously validated for accuracy through the predict function, which assessed their performance on unseen data. Finally, the interpretability of the models was enhanced by analyzing feature importance using the DALEX package, providing insights into which variables significantly influenced the predictions made by each model.

### 2.7. Immunomodulatory Molecule Screening

The expression patterns of immunostimulatory and immunosuppressive genes, along with various chemokines and human leukocyte antigens (HLAs), were meticulously compared between the groups characterized by high and low levels of PAK2. To further investigate the intricate relationships between these expression patterns and immune cell infiltration, correlations were computed using data derived from TIMER 2.0, a comprehensive resource for immune infiltration analysis. The results of these correlations were then visualized in the form of Spearman heatmaps, providing a clear and insightful representation of the associations between the different variables under study.

### 2.8. Drug Repurposing Analysis

Connectivity Map (cMAP) analysis successfully identified a range of compounds that specifically target PAK2, a protein of significant interest in cancer research. To further investigate the relationship between these compounds and PAK2, a gene signature comprising the top 150 genes that were either upregulated or downregulated in tumors characterized by high levels of PAK2 expression was meticulously matched against the extensive cMAP profiles. This matching process utilized the sophisticated XSum algorithm, which calculated similarity scores for a total of 1288 distinct compounds, thereby providing valuable insights into potential therapeutic options that could effectively modulate PAK2 activity in cancer treatment.

### 2.9. Molecular Docking

Compound structure files were obtained from the PubChem Database (https://pubchem.ncbi.nlm.nih.gov/). The 3D modeling and energy minimization of these compounds were performed using Chemdraw version 20.0. Protein structures were retrieved from the AlphaFold Protein Structure Database (https://alphafold.ebi.ac.uk/). The preprocessing of proteins, which involved the removal of water molecules and redundant ligands, as well as the incorporation of hydrogen atoms, was carried out with PyMol 2.4. The outcomes of molecular docking were represented by the binding energies, which were averaged over three independent trials, and the standard deviations were computed. Furthermore, the Human Protein Atlas (HPA) was utilized (http://www.proteinatlas.org/) to verify the expression levels of the validation protein (Supporting Information).

## 3. Results

**Figure 1:**
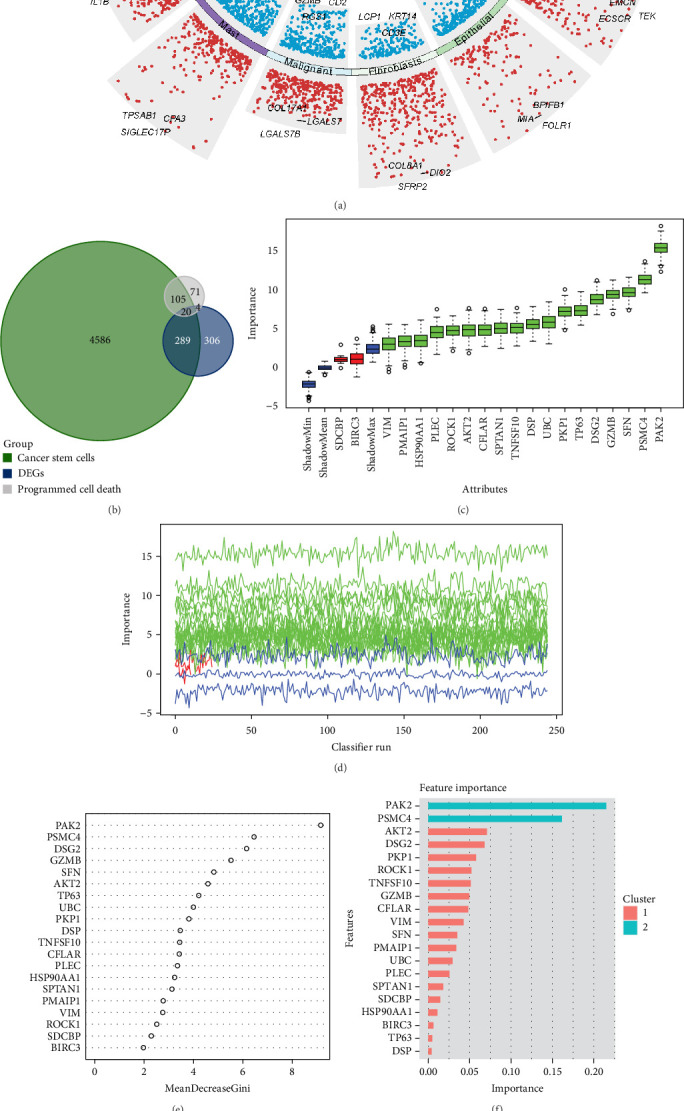
Screening for HNSC key genes associated with programed cell death. (A) Identification of specific markers for various cell types using FindAllMarkers function on GSE150321 dataset. (B) Analysis of the intersection between genes associated with programed cell death and genes associated with cancer stem cells. (C–H) Multiple machine learning algorithms to screen key genes.

We used the FindAllMarkers function on the GSE150321 dataset to identify cell-type-specific markers associated with HNSC pathogenesis, as illustrated in Figure 1A. Following preliminary analysis, we precisely identified 20 target genes by performing an intersection operation between these characteristic genes and those associated with PCD and CSCs (Figure 1B). To further refine our findings, we screened these identified genes using a variety of advanced ML algorithms, which provided us with valuable insights into their significance. Notably, among these genes, PAK2 emerged as the most critical gene across multiple ML algorithms, highlighting its potential importance in the pathology of HNSC. Consequently, we decided to conduct a more in-depth follow-up analysis on PAK2 to explore its implications further (Figure 1C–H).

### 3.1. Clinical Value of PAK2 in HNSC

**Figure 2:**
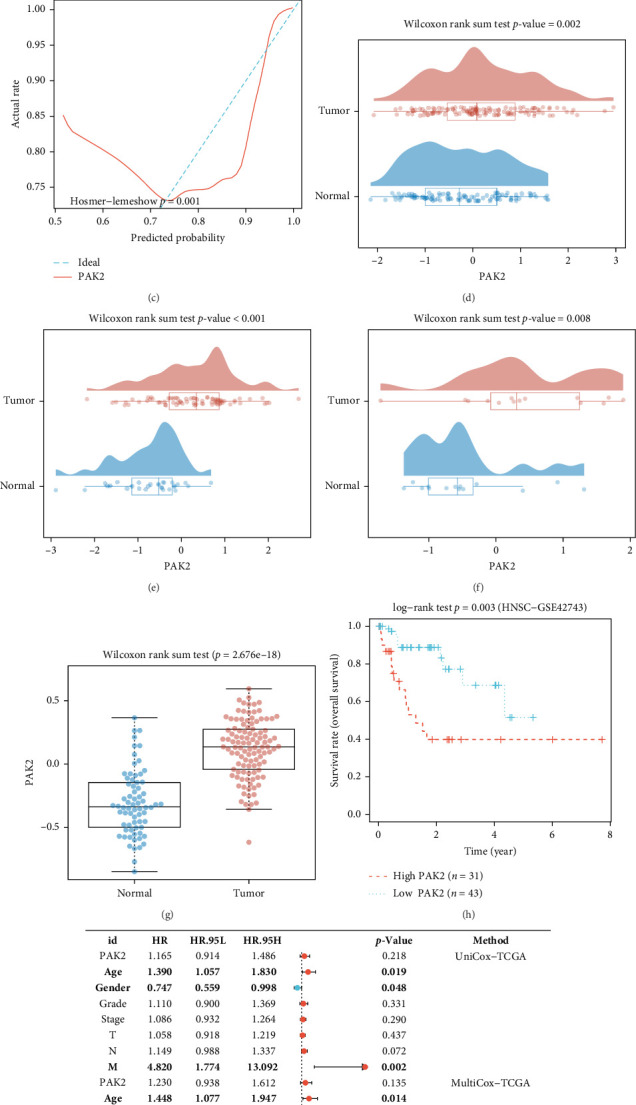
High expression of PAK2 in HNSC and its diagnostic and prognostic value. (A) PAK2 expression was significantly elevated in the tumor. (B) Diagnostic value of PAK2. (C) Correction curve. (D–F) Data from external datasets showed that PAK2 expression was significantly elevated in tumors (GSE42743, GSE75538, E_MTAB_8588). (G) Data from CPTAC dataset showed that PAK2 expression was significantly elevated in tumors. (H, I) KM curves and forest plots indicate that patients with high PAK2 expression have shorter survival.

To determine the clinical significance of PAK2 in HNSC, we analyzed the data in TCGA. The results showed that the expression of PAK2 was significantly elevated in tumors and had strong diagnostic value (Figure 2A, B). Moreover, the calibration curves along with the goodness-of-fit assessments for the predicted outcomes in both tumor and normal cohorts indicated that PAK2 serves as a reliable biomarker for the diagnosis of HNSC. Notably, no significant deviation from an optimal fit was observed, suggesting that the application of PAK2 for determining the tumor status of tissues closely aligns with the theoretical model (Figure 2C). Additionally, analyses of three independent datasets corroborated that the expression levels of PAK2 were markedly increased in tumor samples (Figure 2D–F). This finding was further validated by proteomics data (Figure 2G). Kaplan–Meier survival curves revealed that patients exhibiting elevated PAK2 expression experienced reduced overall survival rates (Figure 2H). The forest plot also indicated the prognostic value of PAK2 (Figure 2I).

### 3.2. Potential Biological Functions of PAK2

**Figure 3:**
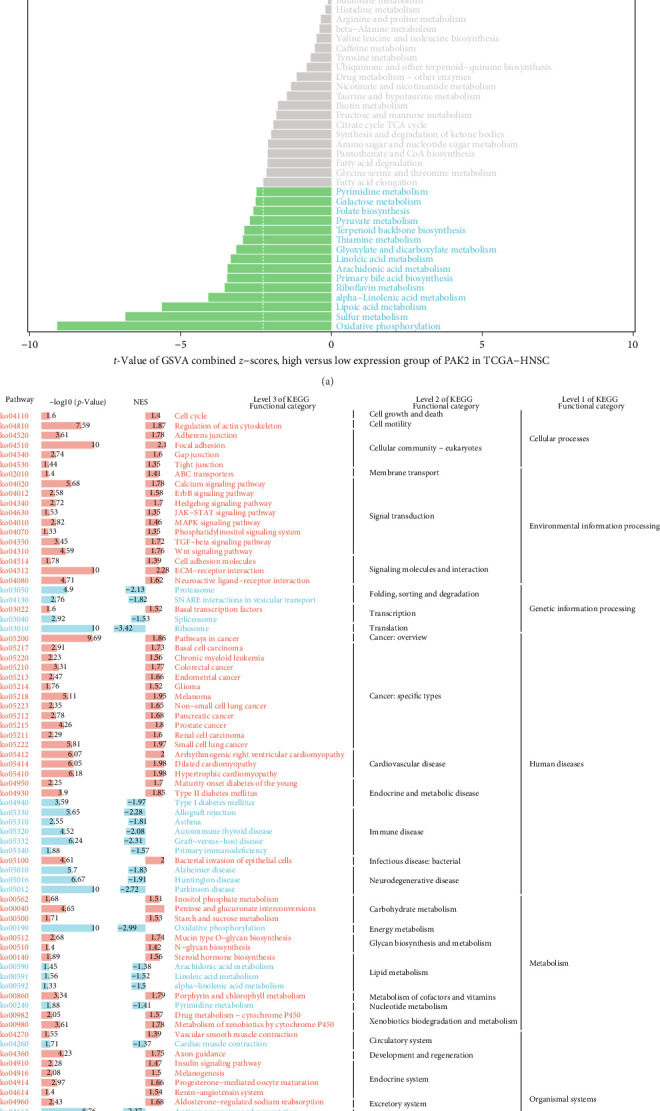
Biological functions of PAK2. (A) Differences in metabolic genomes between high and low expression groups. (B) Enrichment analysis results of the high and low expression groups. (C) Horizontal coordinate is the score of each functional state, vertical coordinate is the amount of PAK2 expression, and R is Pearson correlation analysis.

To understand the role of PAK2 in HNSC, we analyzed the biological functions of PAK2 in detail. We employed the GSVA parameter algorithm from the GSVA package in R to evaluate the metabolic gene set within the KEGG database. The samples were categorized into two groups based on PAK2 gene expression: the high expression group comprised the top 30% of samples exhibiting elevated PAK2 levels, while the low expression group included the bottom 30% with diminished expression. The differences in GSVA scores for the metabolic gene sets between these high and low expression groups were analyzed utilizing the limma software package, as illustrated in Figure 3A. Furthermore, we conducted a genomic enrichment analysis grounded in the KEGG genome for the differentially expressed genes identified in both expression groups, as depicted in Figure 3B. Additionally, we computed the *z*-score for 14 distinct tumor states using GSVA and assessed the Pearson correlation between PAK2 expression and GSVA scores. The findings revealed a significant positive correlation between PAK2 expression and the variables of DIFFERENCE and METASTASIS, closely aligning with the results obtained from the enrichment analysis, as shown in Figure 3C.

### 3.3. Relationship Between PAK2 and Immune Scores

**Figure 4:**
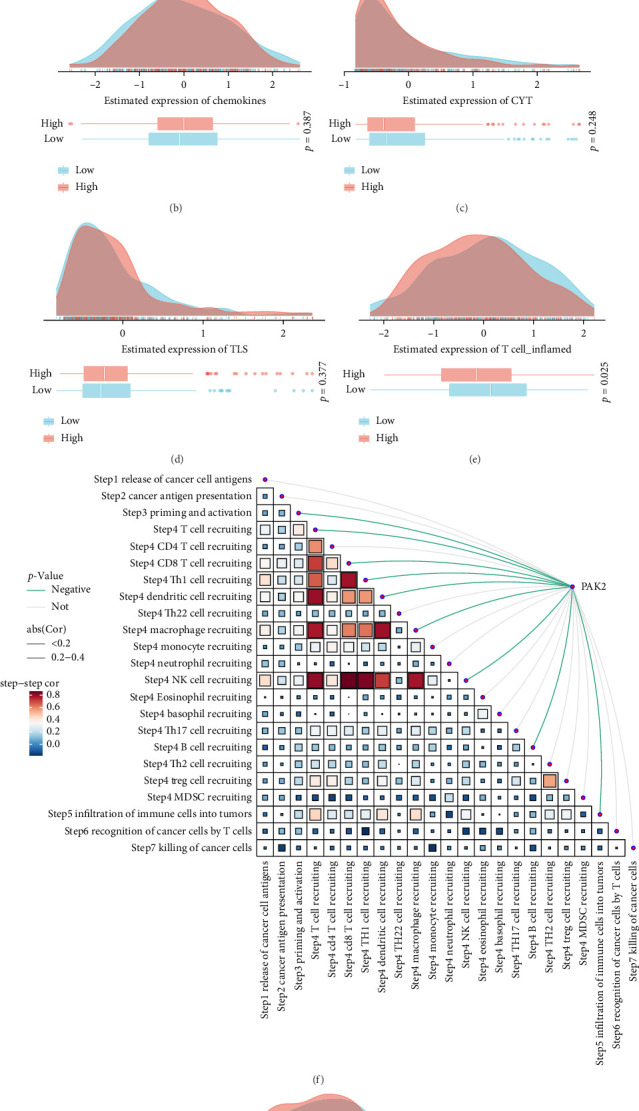
The negative correlation between high PAK2 expression and immune status. (A) Relationship of PAK2 expression with immune infiltration and genomic status based on scores characterizing immunogenicity, DNA damage, etc. (F) Spearman correlation between TIP score and PAK2 expression. (B–G) Differences in the five scores between the high/low PAK2 expression groups.

Patients were stratified into four distinct categories—Q1, Q2, Q3, and Q4—based on quartiles of PAK2 expression levels. The Q1 group comprises the top 25% of samples exhibiting the highest levels of PAK2 expression, whereas Q4 encompasses the lowest 25% with minimal expression. By leveraging findings from previous investigations concerning immune responses and genomic profiles, we computed the mean scores for each of the four patient categories. The accompanying heatmap displays, sequentially from left to right, the average immune response scores alongside genomic status scores for each subtype (Q1, Q2, Q3, and Q4), revealing that the Q1 cohort, marked by elevated PAK2 expression, also recorded higher scores (Figure 4A). Our objective was to scrutinize the interplay between PAK2 expression and the anticancer immune landscape throughout the seven phases of the cancer immune cycle. These phases encompass cancer cell antigen release, presentation of cancer antigens, initiation and activation of the immune response, translocation of immune cells to the tumor site, infiltration of immune cells into the tumor, and the recognition and destruction of cancer cells by T cells. To quantify the scores for each stage across various tumors, we employed tracking immunophenotyping of tumors (tIP). The findings demonstrated a notable negative correlation between PAK2 expression and these immune states (Figure 4F). Additionally, EASY serves as a predictive instrument for biomarker-driven immunotherapies, founded on a model of cancer-specific immune responses, aimed at anticipating antitumor immune responses derived from RNA-seq data. We assessed the differences in five scores—cytolytic activity, tertiary lymphoid structure, interferon-*γ* signature, inflammatory T cells, and chemokines—between the high and low PAK2 expression groups using TCGA-HNSC data (Figure 4B–G).

**Figure 5:**
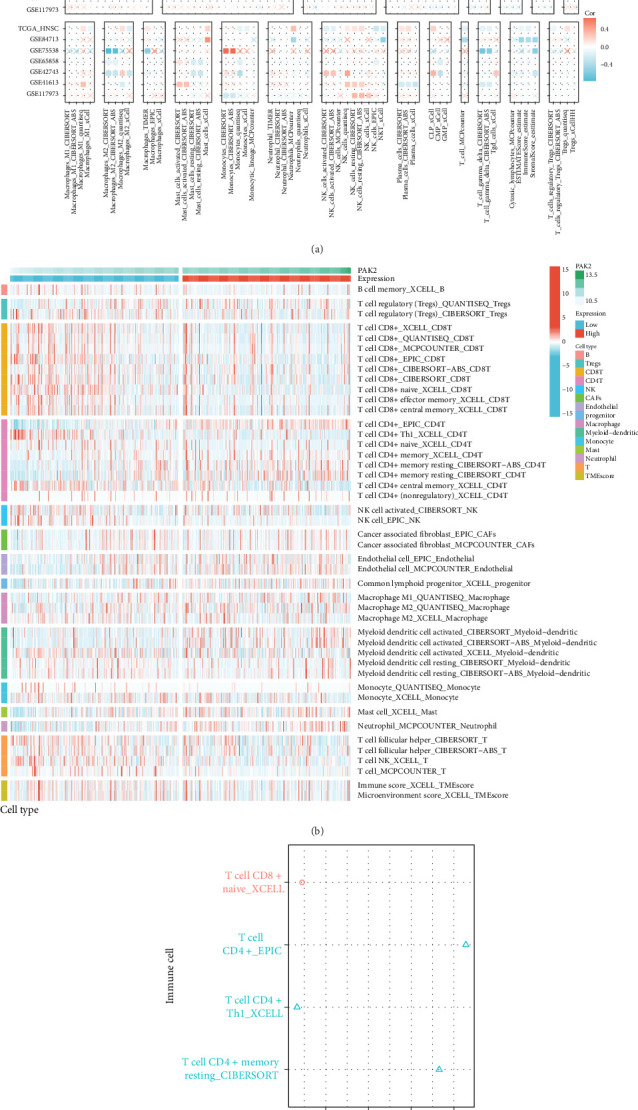
The correlation between PAK2 and immune cells. (A, C) Multiple algorithms to assess the Spearman correlation of PAK2 expression with different immune infiltrating cells in HNSC. The colors of the squares reflect the correlation coefficients (*p*-value less than 0.05), with redder colors representing values closer to 1 (positive correlation) and bluer colors representing values closer to −1 (negative correlation). (B) Immune cell infiltration in PAK2-high and low expression groups.

A comprehensive examination of diverse algorithms can greatly improve our comprehension of the complex mechanisms and unique attributes linked to immune infiltration, thereby shedding light on the intricate pathogenesis of tumors and providing novel strategies for the diagnosis and management of a broad spectrum of diseases. Accordingly, our study sought to clarify the association between PAK2 and immune cells specifically in the realm of HNSC. To facilitate this, we utilized a multialgorithmic strategy to rigorously evaluate the Spearman correlation between PAK2 expression levels and different types of immune infiltrating cells. The findings from our analysis indicated that PAK2 displayed a negative correlation with most immune cell types, as demonstrated in Figure 5A. Additionally, the corresponding heatmap illustrated that the variation in the extent of immune cell infiltration between groups with high and low PAK2 expression levels was relatively slight, as shown in Figure 5B. Notably, PAK2 demonstrated a significant positive correlation with CD4-positive T cells, as shown in Figure 5C, highlighting a potentially crucial interaction that warrants further investigation.

### 3.4. Spatial Transcriptomics Reveals the Role of PAK2

**Figure 6:**
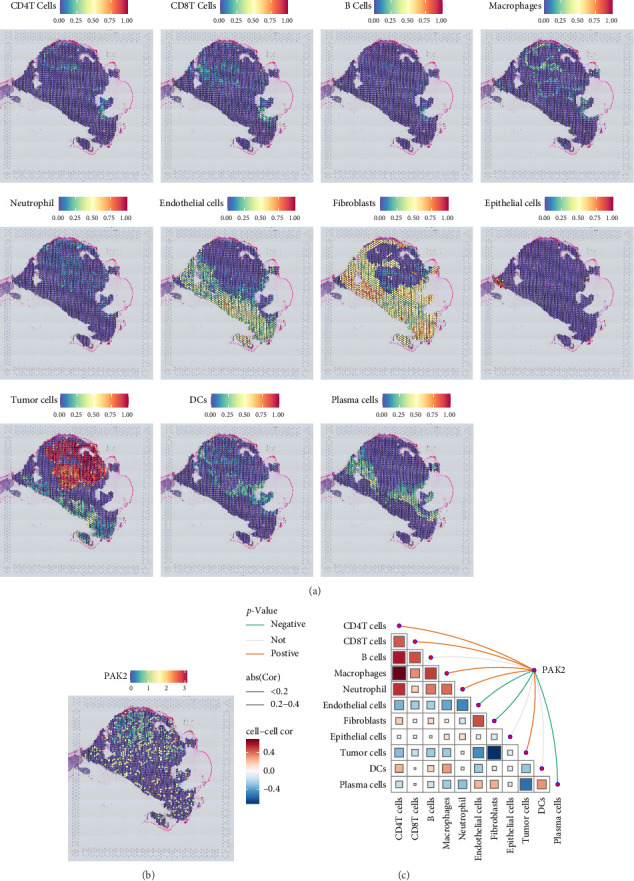
Spatial transcriptomics reveals the role of PAK2. (A) Spatial distribution of various cell types. (B) Expression levels of PAK2 in spatial transcriptome data. (C) Correlation between cell content and PAK2 expression.

Spatial transcriptome sequencing serves as a groundbreaking technique that allows researchers to simultaneously acquire both the spatial location information of individual cells and their corresponding gene expression data. This dual capability provides an invaluable research tool for gaining insights into the intricate functions of tissue cells and the complex interactions occurring within their microenvironments. To illustrate this, we visualized the gene expression landscape across each distinct microregion by utilizing the spatial transcriptome data identified as GSM5494476. In our investigation, we utilized the SpatialFeaturePlot function available in the Seurat package, which facilitated the effective visualization of the enrichment score corresponding to each individual cell type. In this representation, a darker hue indicates a higher enrichment score, which denotes an increased prevalence of the specific cell type within the specified area, as illustrated in Figure 6A. Furthermore, we also visualized the expression levels of the gene PAK2, as shown in Figure 6B, and conducted a thorough analysis to explore the correlation between the calculated cell content and the expression of PAK2 across all spots, which is illustrated in Figure 6C.

### 3.5. Analysis of PAK2 in Single-Cell Data

**Figure 7:**
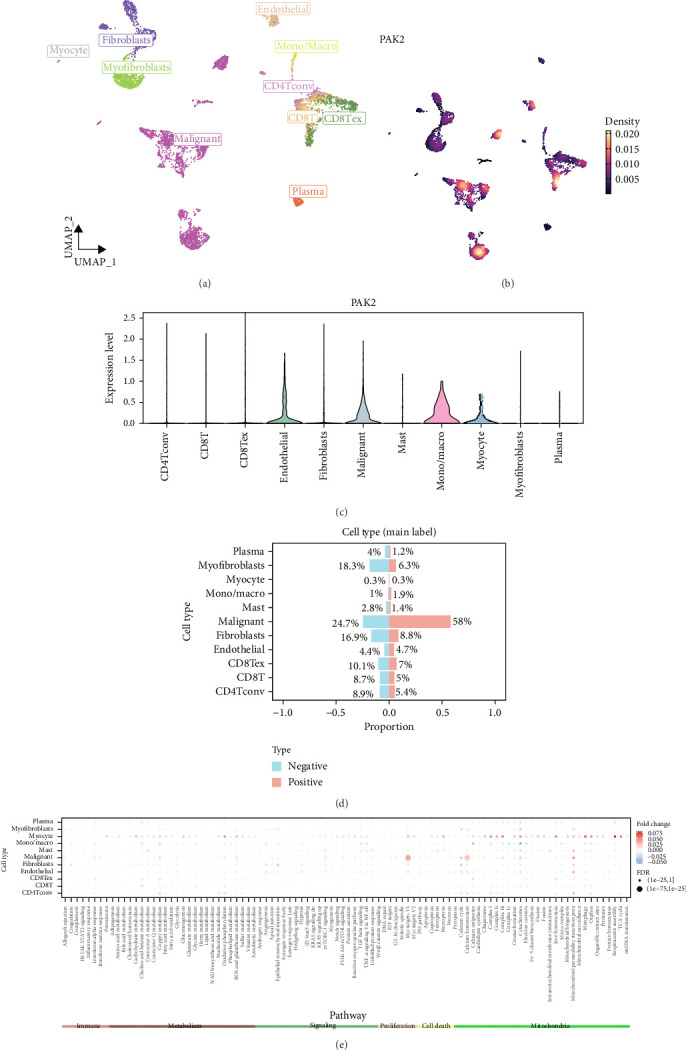
Analysis of PAK2 in single-cell data. (A) Single-cell data distribution after dimensionality reduction of the GSE103322 dataset. (B) Expression levels of PAK2 in single cells. (C) Differences in PAK2 expression in different cell types. (D) Proportion of each cell type in the expression-positive and expression-negative groups. (E) Difference in scores between PAK2 expression-negative and PAK2 expression-positive groups.

UMAP effectively illustrated the distribution of single-cell data following the dimensionality reduction of the GSE103322 dataset, successfully distinguishing various cell types based on their distinct expression profiles (Figure 7A). We visualized the expression levels of PAK2 in single cells (Figure 7B). We evaluated variations in PAK2 expression across distinct cell types (Figure 7C). Cells were classified into two groups: expression-positive and expression-negative, based on the presence or absence of PAK2 expression. Subsequently, we calculated the proportions of each cell type within both the expression-positive and expression-negative categories (Figure 7D). Furthermore, we analyzed the scores associated with immune responses, metabolic processes, signaling pathways, cellular proliferation, apoptosis, and mitochondrial-related biological pathways, and compared the score disparities between the PAK2 expression-negative and PAK2 expression-positive groups (Figure 7E).

Variations in the expression levels of immune-related molecules between groups exhibiting high and low expression of PAK2.

**Figure 8:**
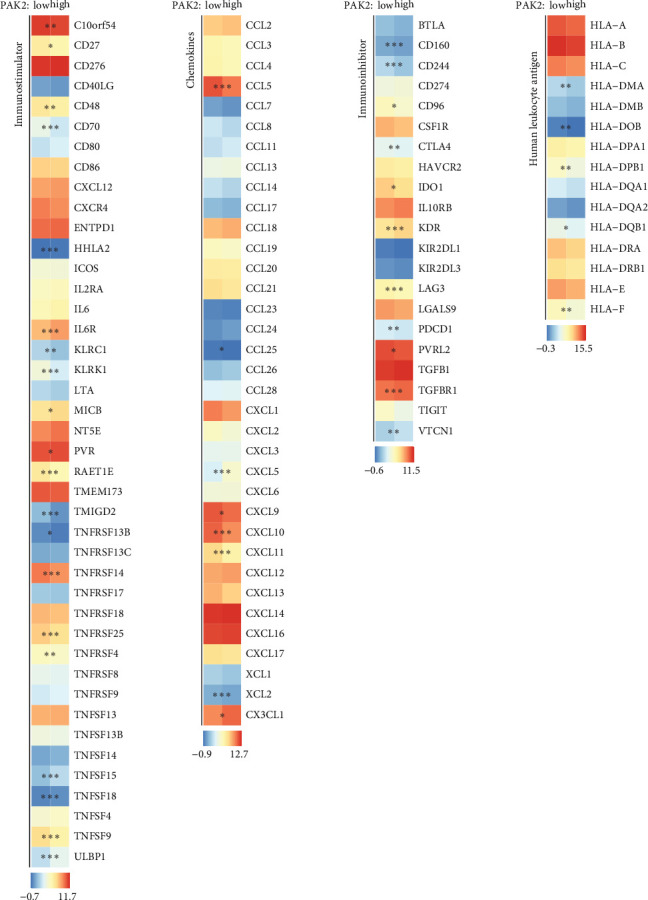
Differences in the expression of immunostimulatory genes, immunosuppressive genes, chemokine-related genes, and human leukocyte antigen-related genes in PAK2-high/low expression groups. Statistical significance is indicated as follows: *⁣*^*∗*^*p* < 0.05, *⁣*^*∗∗*^*p* < 0.01, and *⁣*^*∗∗∗*^*p* < 0.001.

To further understand the intricate relationship between immune-related molecules and the protein PAK2, we conducted a comprehensive exploration of the differences in the expression levels of four distinct types of genes: immunostimulatory genes, which play a crucial role in activating immune responses; immunosuppressive genes, which are involved in downregulating immune activity; chemokines, which are essential for directing the movement of immune cells; and HLAs, which are vital for the recognition of self versus nonself by the immune system. This investigation was specifically focused on comparing the expression profiles within two groups categorized by their levels of PAK2 expression—those with high levels of PAK2 and those with low levels of PAK2 expression, as illustrated in Figure 8.

### 3.6. Relationship Between PAK2 and Immunomodulators

**Figure 9:**
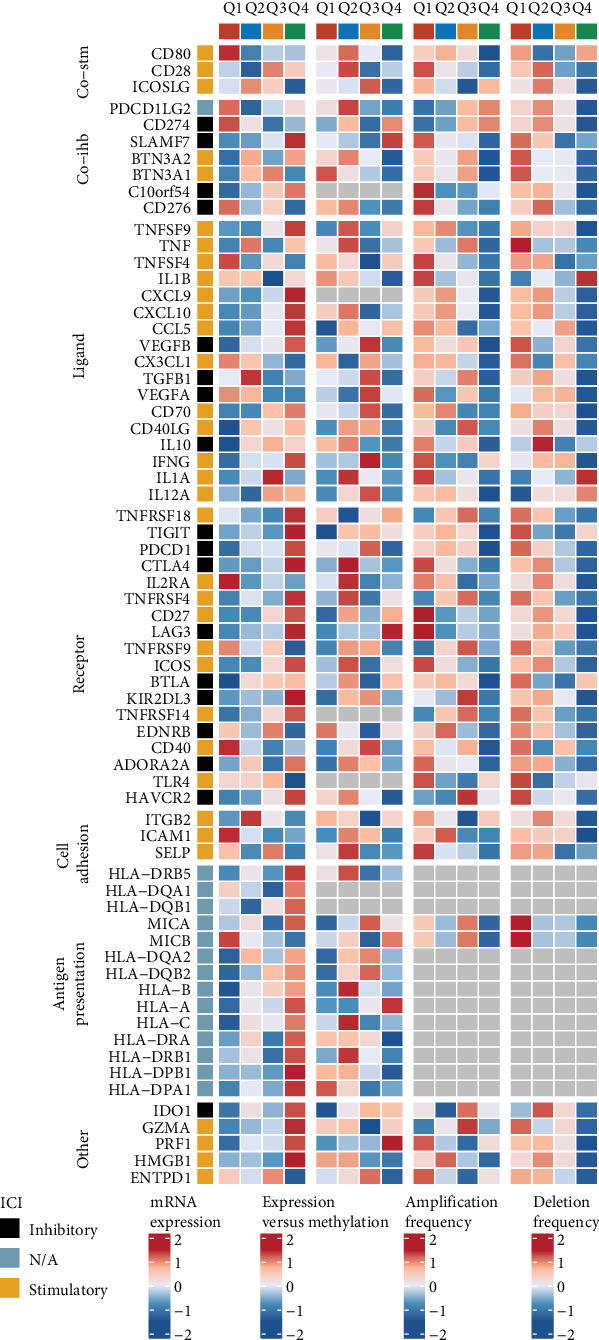
Relationship between PAK2 and immunomodulators.

In addition to our previous investigations, we delved deeply into the intricate and complex relationship between PAK2 and various immunomodulators, meticulously examining how these multifaceted interactions influence immune responses in a variety of contexts. Consequently, we gained a comprehensive understanding of their expression levels and regulatory control patterns across different states of PAK2, as clearly illustrated in Figure 9, which provides a visual representation of our findings. All these findings collectively suggest that PAK2 plays a crucial and multifaceted role in the regulation of the immune system, highlighting its potential significance not only in basic immunological research but also in the development of innovative therapeutic applications aimed at modulating immune responses for better health outcomes.

### 3.7. Targeted Drugs Against PAK2 and Immunohistochemical Results

**Figure 10:**
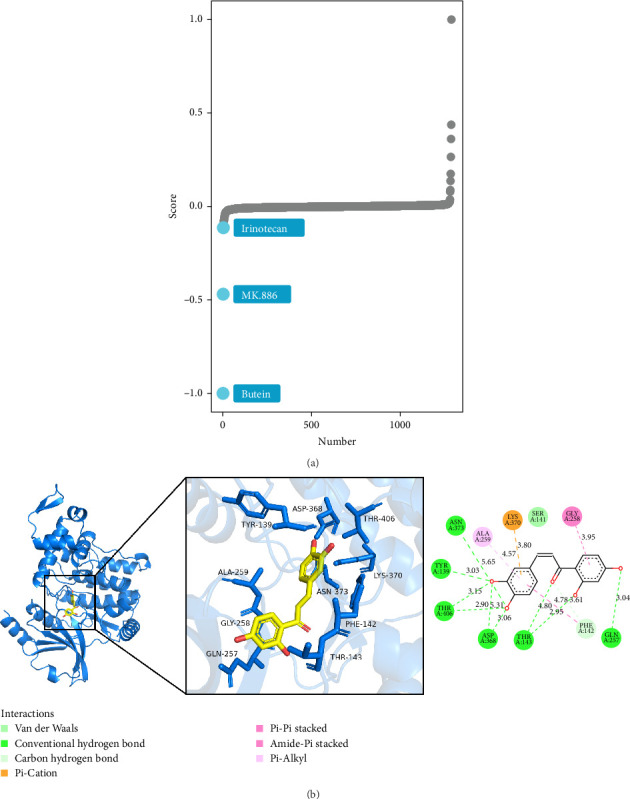
Targeting drugs for PAK2. (A) Exploration of potential therapeutic options that could counteract PAK2-mediated tumor promotion by cMAP analysis. (B) Molecular docking indicates that butein binds efficiently to PAK2.

**Figure 11:**
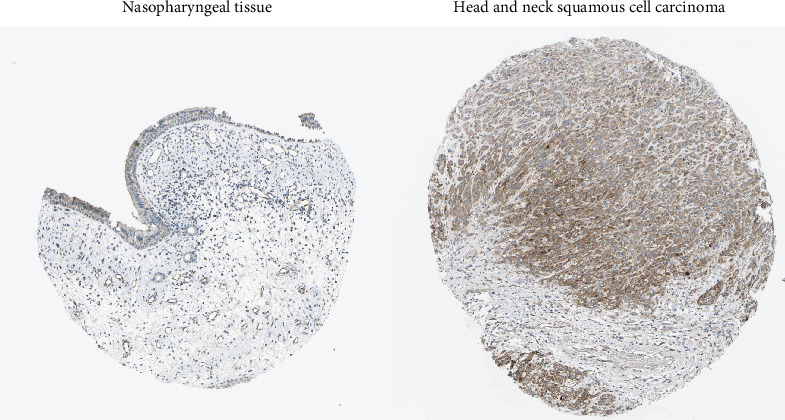
Representative IHC micrographs of PAK2 expression in nasopharyngeal samples and HNSCC from HPA database.

In order to investigate possible therapeutic strategies that might mitigate PAK2-driven tumor promotion, we conducted a cMAP analysis. By contrasting tumor samples from patients exhibiting high versus low expression levels of specific genes, we developed a gene-associated signature encompassing the 150 genes with the most significant upregulation and the 150 genes with the most considerable downregulation. Subsequently, we employed the optimal feature matching technique known as eXtreme Sum (XSum) to compare the gene-associated characteristics against the cMAP gene features, thereby generating similarity scores for all tested compounds. The results showed that butein reversed the molecular signature caused by dysregulation of PAK2 expression, thereby counteracting the PAK2-mediated pro-oncogenic effects (Figure 10A). Molecular docking also showed a good binding interaction between butein and PAK2, and this interaction was also reliable. The docking of butein with PAK2 is shown (Figure 10B). To verify that the protein expression of PAK2 is elevated in HNSCC compared to normal nasopharyngeal tissue, we examined the immunohistochemical findings from the HPA database and gathered clinical samples for further immunohistochemical investigations. The findings indicated a markedly higher protein expression level in HNSCC tissues relative to that observed in normal breast tissue. A representative micrograph of the immunohistochemistry (IHC) results is presented in Figure 11. This study convincingly illustrated the pronounced overexpression of PAK2 in HNSCC tissues, thereby providing substantial evidence for its prospective role as a therapeutic target in the treatment of HNSCC.

## 4. Discussion

This study establishes PAK2 as a central orchestrator of PCD dysregulation in HNSC through multi-omics integration and ML, underscoring the transformative power of systems biology in decoding complex oncogenic networks [20, 21]. The convergence of bulk transcriptomics, scRNA-seq, spatial transcriptomics, and proteomics has enabled a multidimensional dissection of PAK2's role in HNSC pathogenesis—a feat unattainable through reductionist approaches [22, 23]. Traditional single-omics studies, while valuable, often fail to capture the spatial heterogeneity and cellular crosstalk driving PCD resistance [24–26]. For instance, bulk RNA-seq alone might identify PAK2 as differentially expressed but would miss its cell type-specific regulation revealed by scRNA-seq [27, 28]. Similarly, spatial transcriptomics localized PAK2 to immune-excluded niches, a finding invisible to conventional sequencing, thereby linking its expression to geographic immunosuppression [29, 30].

Using the FindAllMarkers function on the GSE150321 dataset, we meticulously identified a total of 20 genes that intersected with programed death-related genes, revealing a fascinating intersection of biological pathways. Among these genes, PAK2 emerged as the most critical and noteworthy gene across multiple ML algorithms, underscoring its potential significance in the context of HNSC. This observation was further bolstered by our subsequent analyses, which compellingly demonstrated that PAK2 expression is significantly elevated in HNSC tumors when compared to normal tissues, suggesting its promising potential as a diagnostic biomarker that could aid in the early detection and treatment of this malignancy [31].

To elucidate the intricate biological functions of PAK2 in HNSC, we meticulously performed GSVA alongside comprehensive genomic enrichment analysis. Our results compellingly demonstrated that the expression levels of PAK2 are positively correlated with various metabolic pathways that are intricately linked to both cell differentiation and the process of metastasis. This correlation suggests that PAK2 may play a pivotal role in promoting tumor progression by effectively modulating these critical pathways. Consequently, these findings provide a robust molecular basis for a deeper understanding of the significant role that PAK2 plays in the pathogenesis of HNSC, potentially opening avenues for targeted therapeutic strategies in the future [32, 33]. This suggests that PAK2 may be a key molecule driving the acquisition and maintenance of CSC properties in HNSC cells, thereby contributing to tumor invasion, metastasis, and recurrence.

Analysis of TCGA data revealed that PAK2 is not only overexpressed in HNSC tumors but also exhibits significant diagnostic value. Calibration curves and goodness-of-fit tests confirmed PAK2 as a reliable predictor for HNSC diagnosis. Furthermore, consistent results from three external datasets and proteomics data demonstrated elevated PAK2 expression in tumor tissues, further supporting its diagnostic potential. Survival analysis based on KM curves and forest plots indicates that patients with high PAK2 expression exhibit shorter overall survival, suggesting PAK2 may also possess potential as a prognostic biomarker for HNSC. The synergy of ML and multi-omics was pivotal in prioritizing PAK2 from 20 PCD-related candidates. While classical PCD genes are well-characterized at the genomic level, our integrative framework exposed PAK2's unique dominance across algorithms (LASSO, SVM, and RF), suggesting its multimodal influence spanning genomic, metabolic, and immune axes. Proteomic validation further confirmed PAK2's translational relevance, bridging the gap between mRNA abundance and functional protein activity—a critical step often overlooked in transcriptome-centric studies [34, 35].

Through comprehensive analysis of the complex dynamics of immune scores and immune cell infiltration, we identified a significant trend: PAK2 expression exhibits a marked negative correlation with the overall anticancer immune state. Specifically, PAK2 expression exhibited negative correlations across multiple critical steps of the cancer immune cycle, including the release of cancer antigens, antigen presentation, and the infiltration of immune cells into the tumor microenvironment. Furthermore, while PAK2 expression was negatively correlated with most immune infiltrating cell types, it notably showed a positive correlation with CD4+ T cells. These intriguing findings strongly suggest that PAK2 may play a pivotal role in modulating the tumor immune microenvironment, potentially facilitating immune evasion and contributing to the progression of the tumor [36, 37].

Finally, the cMAP-driven drug discovery process serves as a compelling example of how the integration of diverse multi-omics data can directly inform and significantly enhance therapeutic strategies in the realm of modern medicine. By meticulously combining PAK2-associated gene signatures with extensive and comprehensive chemical perturbation databases, we successfully identified butein—a natural compound renowned for its various beneficial properties—as a potent PAK2 inhibitor, effectively circumventing the numerous limitations that are often encountered in traditional target-centric drug screens that can restrict the discovery of novel therapeutics. This innovative and forward-thinking approach closely mirrors the recent successes that have been observed in the rapidly evolving field of network pharmacology, where multi-omics-derived hubs have yielded clinically actionable targets that hold the potential to profoundly impact patient treatment and outcomes in a meaningful way.

## 5. Conclusion

Our study exemplifies how multi-omics integration transcends the sum of its parts, transforming PAK2 from a candidate gene into a context-aware therapeutic node governing PCD, metabolism, and immunity. This holistic framework not only advances HNSC biology but also provides a blueprint for studying other oncogenic checkpoints in the multi-omics era.

## Data Availability

The data that support the findings of this study are available from the corresponding author upon reasonable request.

## Supporting Information

Supporting Information Original data.
