# Supplementary figures and images for: Discovery of PAK2 as a Key Regulator of Cancer Stem Cell in Head and Neck Squamous Cell Carcinoma Using Multi-Omic Techniques
Source: Stem Cells Int. 2025 Nov 19;2025:1325262. doi: 10.1155/sci/1325262 (PMC12657082; doi:10.1155/sci/1325262)

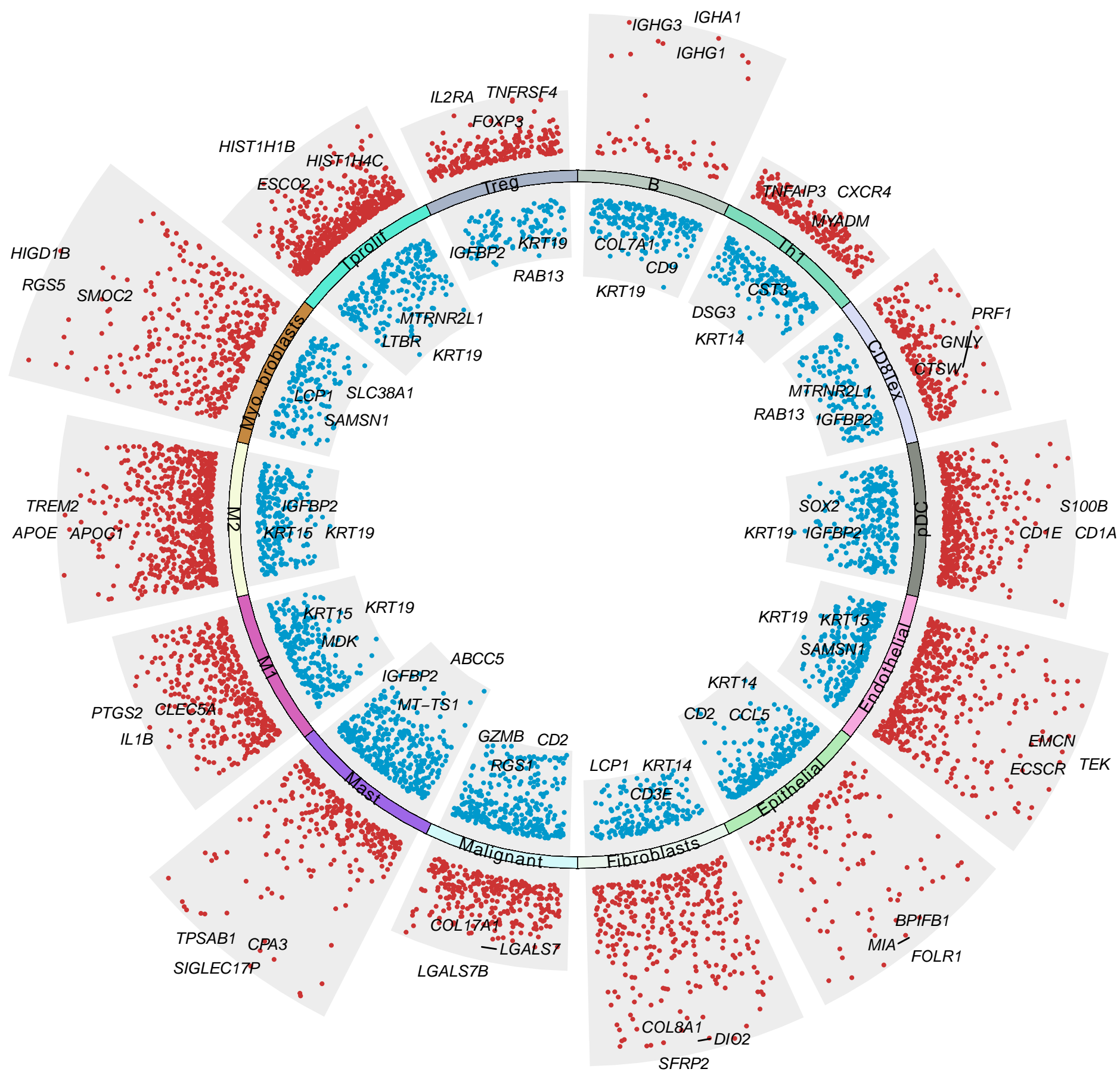

Supplement: Supporting Information — Original data. [file 1325262.f1.zip › vene/cell.Markers.pdf]

Programmed cell death

DEGs

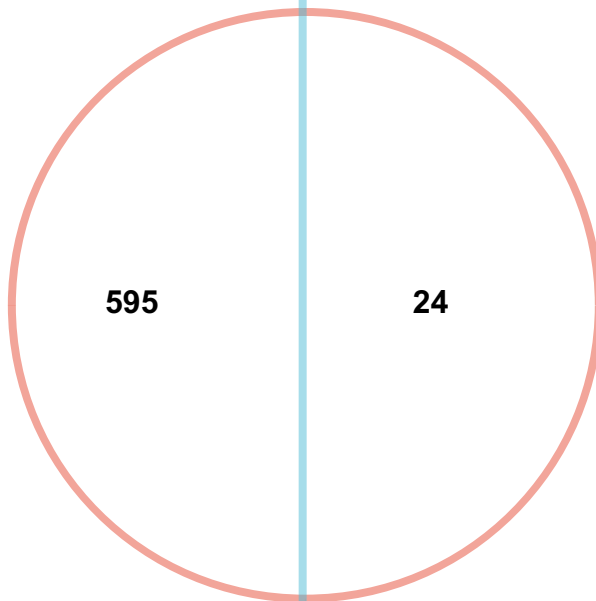

Supplement: Supporting Information — Original data. [file 1325262.f1.zip › vene/Vene.pdf]
