# Supplementary material for: Discovery of PAK2 as a Key Regulator of Cancer Stem Cell in Head and Neck Squamous Cell Carcinoma Using Multi-Omic Techniques
Source: Stem Cells Int. 2025 Nov 19;2025:1325262. doi: 10.1155/sci/1325262 (PMC12657082; doi:10.1155/sci/1325262)
Supplement: Supporting Information — Original data. [file 1325262.f1.zip › vene/Boruta.zip!/ML.Boruta_A.pdf]

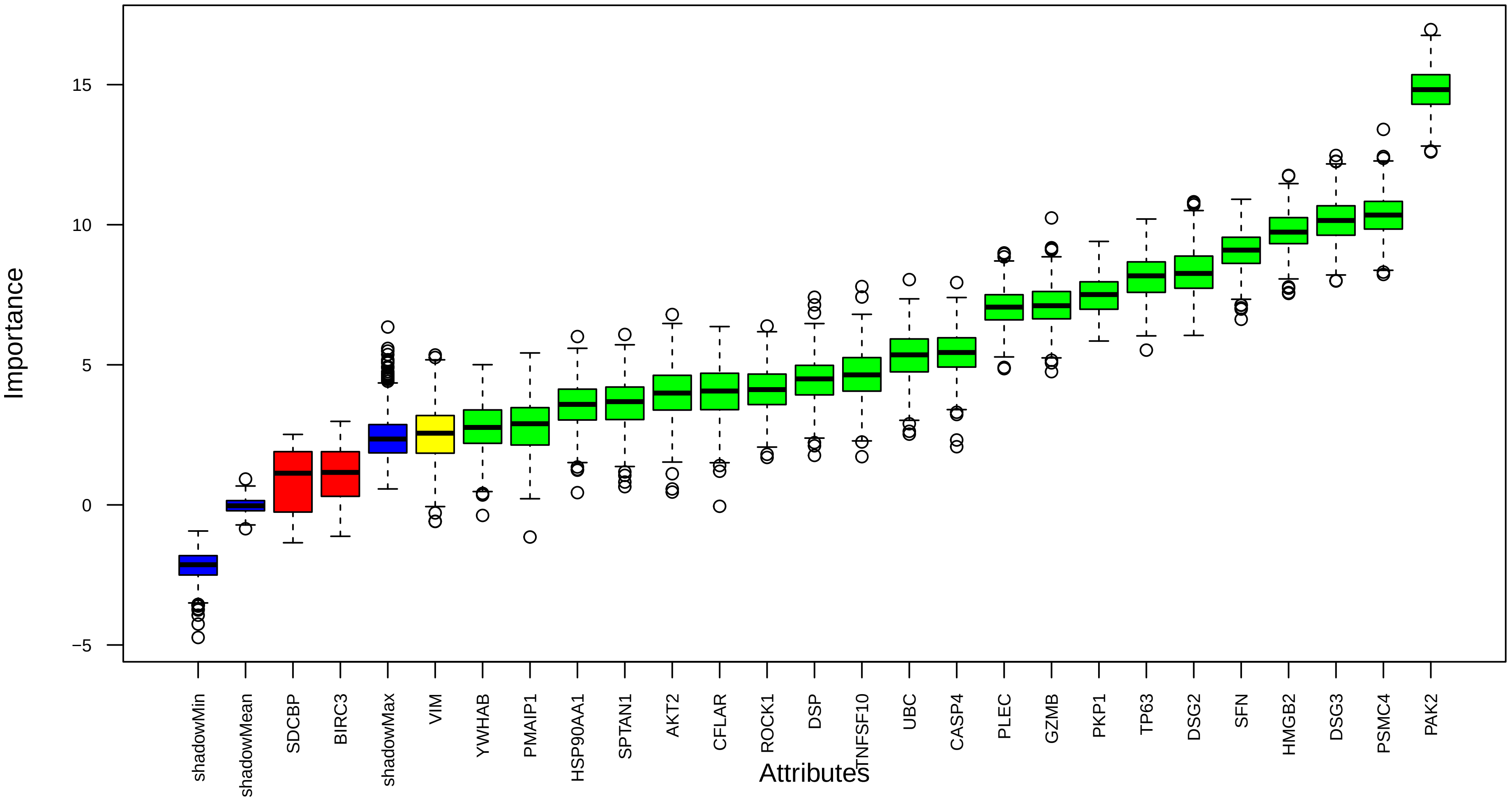

Importance
15
10
5
0
−5
shadowMin
shadowMean
SDCBP
BIRC3
shadowMax
VIM
YWHAB
PMAIP1
HSP90AA1
SPTAN1
AKT2
CFLAR
ROCK1
DSP
TNFSF10
UBC
CASP4
PLEC
GZMB
PKP1
TP63
DSG2
SFN
HMGB2
DSG3
PSMC4
PAK2
Attributes
