# Supplementary material for: Discovery of PAK2 as a Key Regulator of Cancer Stem Cell in Head and Neck Squamous Cell Carcinoma Using Multi-Omic Techniques
Source: Stem Cells Int. 2025 Nov 19;2025:1325262. doi: 10.1155/sci/1325262 (PMC12657082; doi:10.1155/sci/1325262)
Supplement: Supporting Information — Original data. [file 1325262.f1.zip › vene/Lasso.zip!/lasso.cvfit.pdf]

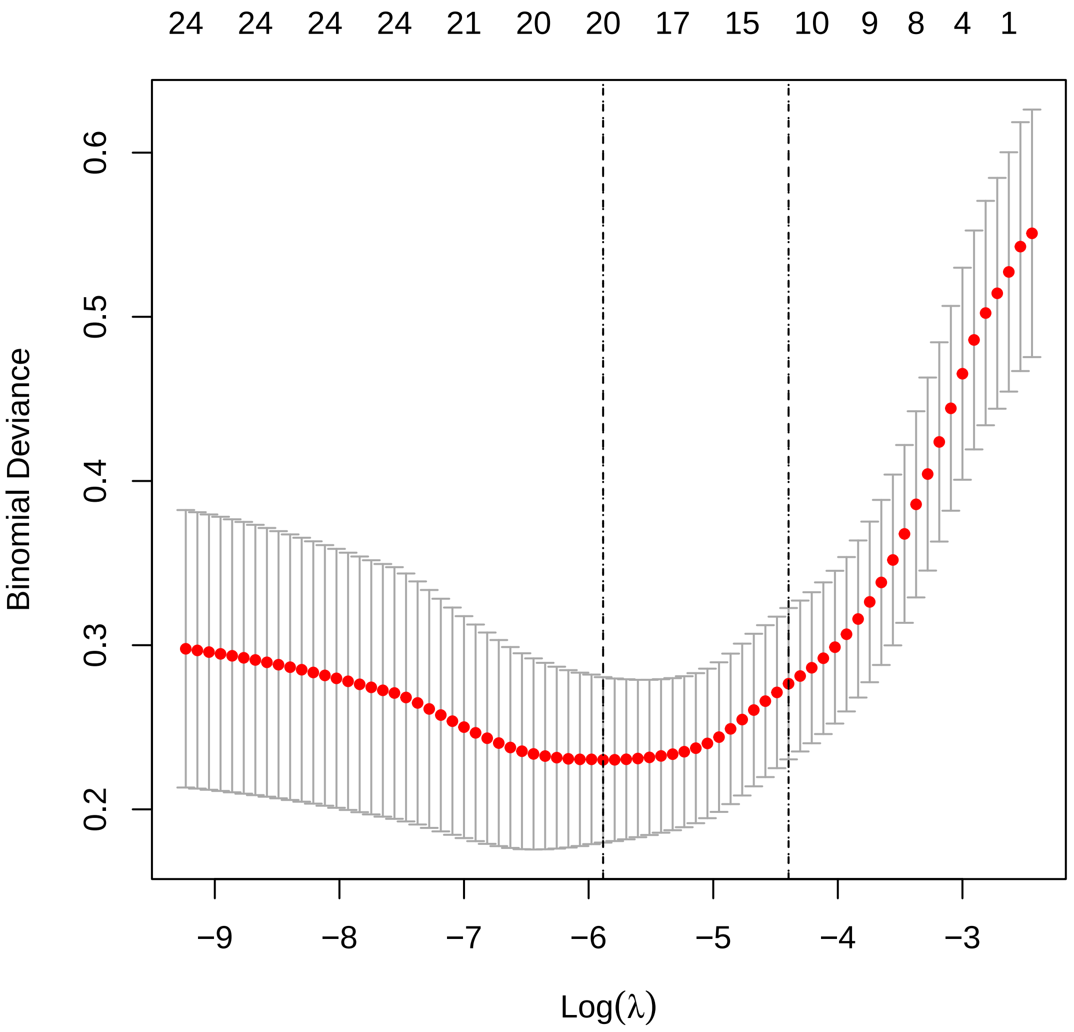

24 24 24 24 21 20 20 17 15 10 9 8 4 1
Binomial Deviance
0.2
0.3
0.4
0.5
0.6
−9 −8 −7 −6 −5 −4 −3
Log(λ)
