# Supplementary material for: Discovery of PAK2 as a Key Regulator of Cancer Stem Cell in Head and Neck Squamous Cell Carcinoma Using Multi-Omic Techniques
Source: Stem Cells Int. 2025 Nov 19;2025:1325262. doi: 10.1155/sci/1325262 (PMC12657082; doi:10.1155/sci/1325262)
Supplement: Supporting Information — Original data. [file 1325262.f1.zip › vene/Lasso.zip!/lasso.pdf]

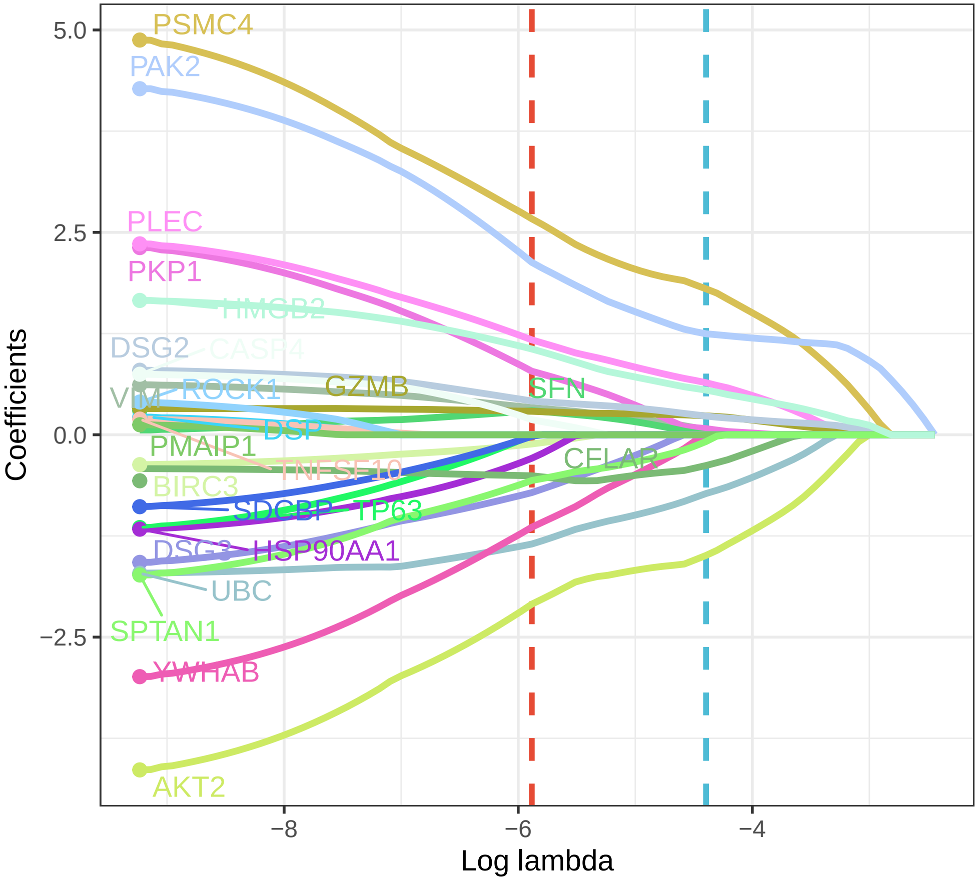

PSMC4
PAK2
PLEC
PKP1
HMGB2
DSG2
CASP4
ROCK1
GZMB
VIM
SFN
DSP
PMAIP1
TNFSF10
CFLAR
BIRC3
SDCBP
TP63
DSG3
HSP90AA1
UBC
SPTAN1
YWHAB
AKT2
5.0
2.5
0.0
−2.5
−8
−6
−4
Coefficients
Log lambda
