# Supplementary material for: Discovery of PAK2 as a Key Regulator of Cancer Stem Cell in Head and Neck Squamous Cell Carcinoma Using Multi-Omic Techniques
Source: Stem Cells Int. 2025 Nov 19;2025:1325262. doi: 10.1155/sci/1325262 (PMC12657082; doi:10.1155/sci/1325262)
Supplement: Supporting Information — Original data. [file 1325262.f1.zip › vene/RF.zip!/Randomforest_A.pdf]

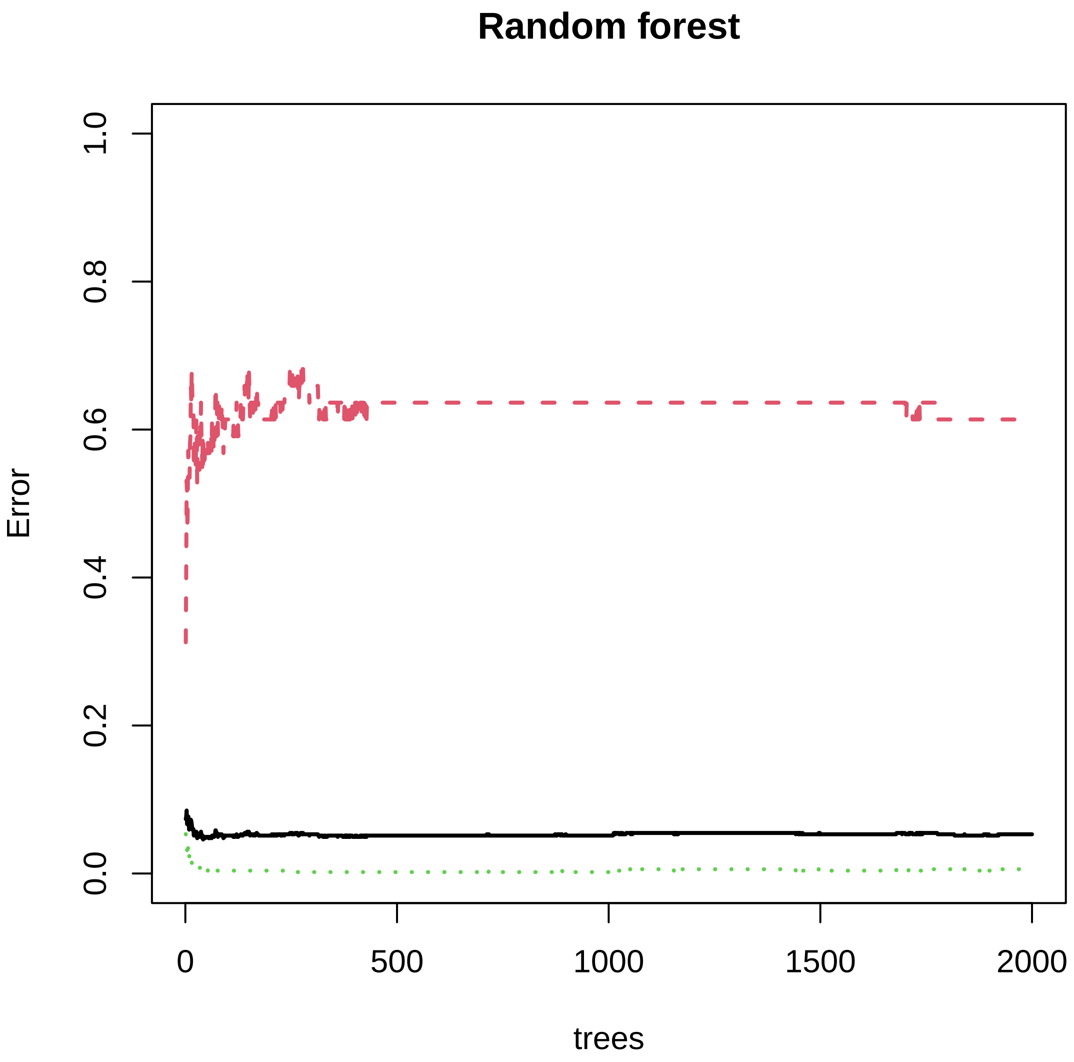
Random forest
Error
trees
0.0
0.2
0.4
0.6
0.8
1.0
0
500
1000
1500
2000
