# Supplementary material for: Discovery of PAK2 as a Key Regulator of Cancer Stem Cell in Head and Neck Squamous Cell Carcinoma Using Multi-Omic Techniques
Source: Stem Cells Int. 2025 Nov 19;2025:1325262. doi: 10.1155/sci/1325262 (PMC12657082; doi:10.1155/sci/1325262)
Supplement: Supporting Information — Original data. [file 1325262.f1.zip › vene/RF.zip!/Randomforest_geneImportance.pdf]

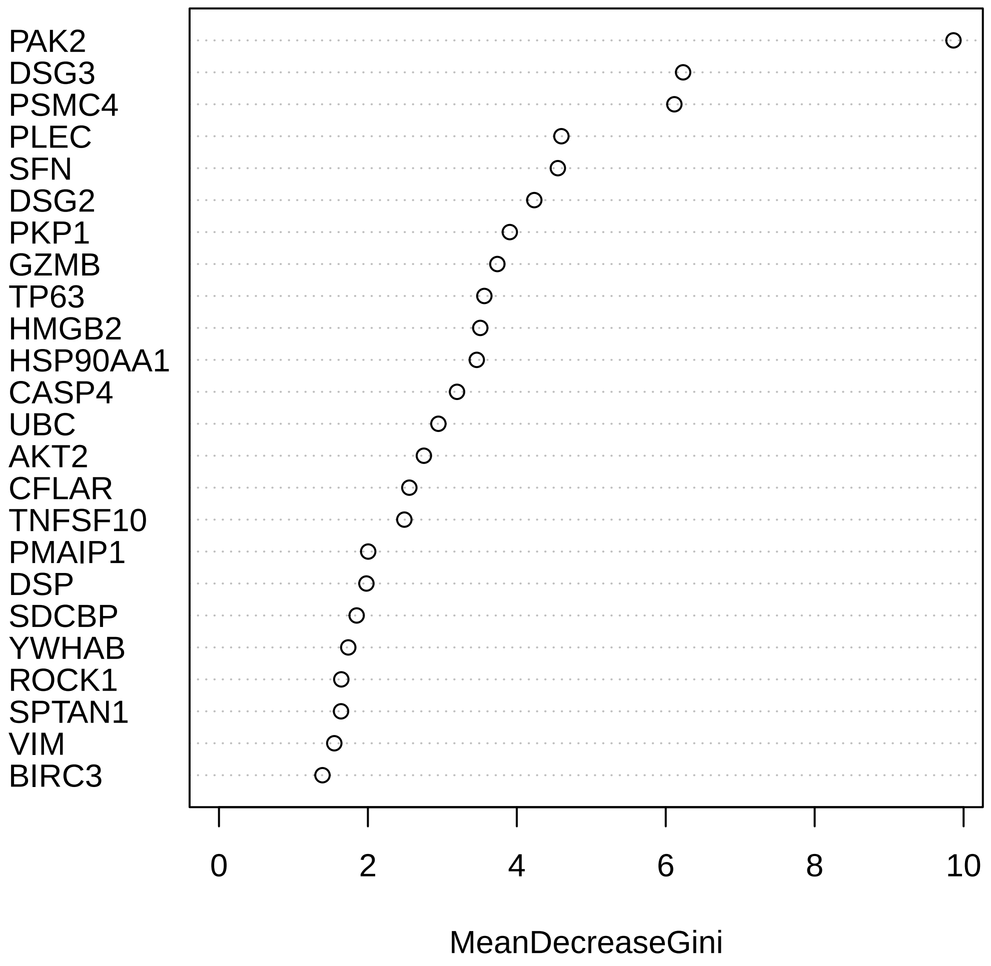

PAK2
DSG3
PSMC4
PLEC
SFN
DSG2
PKP1
GZMB
TP63
HMGB2
HSP90AA1
CASP4
UBC
AKT2
CFLAR
TNFSF10
PMAIP1
DSP
SDCBP
YWHAB
ROCK1
SPTAN1
VIM
BIRC3
0
2
4
6
8
10
MeanDecreaseGini
