# Supplementary material for: Discovery of PAK2 as a Key Regulator of Cancer Stem Cell in Head and Neck Squamous Cell Carcinoma Using Multi-Omic Techniques
Source: Stem Cells Int. 2025 Nov 19;2025:1325262. doi: 10.1155/sci/1325262 (PMC12657082; doi:10.1155/sci/1325262)
Supplement: Supporting Information — Original data. [file 1325262.f1.zip › vene/xgboost.zip!/XGBoost.pdf]

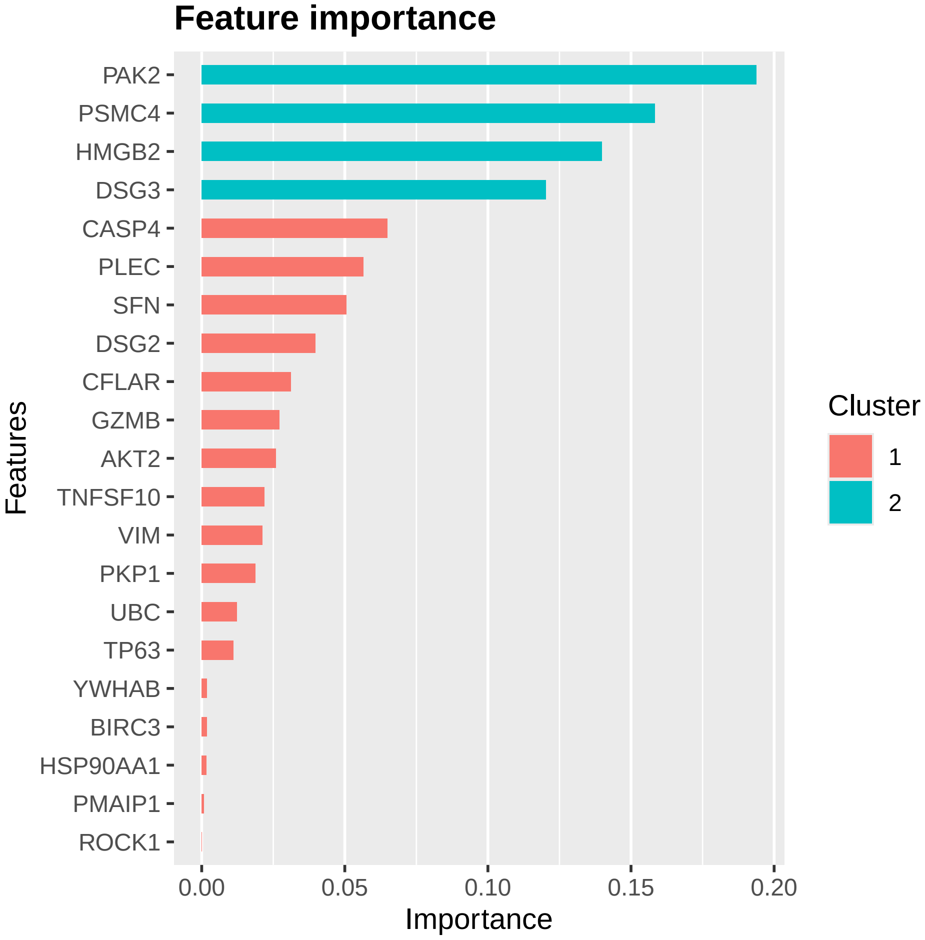
Feature importance
PAK2
PSMC4
HMGB2
DSG3
CASP4
PLEC
SFN
DSG2
CFLAR
GZMB
AKT2
TNFSF10
VIM
PKP1
UBC
TP63
YWHAB
BIRC3
HSP90AA1
PMAIP1
ROCK1
Features
0.00
0.05
0.10
0.15
0.20
Importance
Cluster
1
2
